# Idiopathic pulmonary fibrosis – clinical management guided by the evidence-based GRADE approach: what arguments can be made against transparency in guideline development?

**DOI:** 10.1186/s12916-016-0563-0

**Published:** 2016-02-10

**Authors:** Bram Rochwerg, Holger J. Schünemann, Ganesh Raghu

**Affiliations:** Department of Medicine, McMaster University, Hamilton, ON Canada; Department of Clinical Epidemiology & Biostatistics, McMaster University, Hamilton, ON Canada; Department of Medicine (Division of Pulmonary and Critical Care Medicine), University of Washington, Seattle, WA USA; Center for Interstitial lung Diseases, UW Medical Center, Seattle, WA USA

**Keywords:** Evidence-based, Guideline, Idiopathic pulmonary fibrosis

## Abstract

Evidence-based guidelines have undergone an incredible transformation over the last number of years. Significant advances include explicit linkages of systematic evidence summaries to the strength and direction of recommendations, consideration of all patient-important factors, transparent reporting of the recommendation generation process including conflict of interest management strategies and the production of clinical practice guidelines which use simple and clear language. The Grading of Recommendations Assessment, Development and Evaluation (GRADE) methodology provides a framework for guideline development and was employed to produce the recently published ATS/ERS/JRS/ALAT update on treatment for idiopathic pulmonary fibrosis (IPF). Herein we discuss the advantages of using an evidence-based approach for guideline development using the IPF process and resultant document as an example.

## Background

Evidence-based guidelines have undergone intense evolution over the last 15 years [1–4]. The main driver behind this transformation has been the change in focus from what used to be called expert- or consensus-based to evidence-driven recommendations. This distinction represents a typical misunderstanding as even in the era of evidence-based guidelines, recommendations are developed by clinical experts in the field and require consensus of panel members on the best possible treatment options. It is the transparent link between the evidence and the recommendations and the requirement of making structured expert judgments that represents a shift in the guideline development paradigm. This is true even for evidence-based guidelines that focus on complex and rare diseases such as idiopathic pulmonary fibrosis (IPF).

In 2000, a selected panel of international experts in the field of interstitial lung diseases provided a guideline for the diagnosis and management of IPF [5]. Due to the paucity of evidence available and the relatively immature field of guideline methodology, this document was developed using the conventional consensus-approach based on the opinions of the few expert panel members without a systematic review of the literature or formal quality of evidence evaluation. Regardless, this document, then considered state of the art for the disease, provided useful direction to clinicians in diagnosing and managing patients with IPF. Over the next decade, an increasing number of studies in the field of IPF were published based on this guidance document. With accumulating evidence, it became onerous for practicing clinicians to carefully review and interpret the most current studies. In order to address this challenge and to improve on the previous document, the 2011 guideline document employed an evidence-based approach, namely the GRADE (Grading of Recommendations Assessment, Development and Evaluation) approach to guideline development [6, 7]. The 2011 guideline redefined IPF with precise diagnostic criteria based on clinical, radiological and histopathological features. Also, for the first time in the field of IPF, it provided evidence-based treatment recommendations [8].

In this commentary, we will discuss the motivation behind this evolution in evidence-based guidelines and the benefits of developing guidelines that are linked to underlying evidence summaries along with explicit assessments of the quality (certainty) of the evidence. We will focus on the utility of GRADE methodology [7, 9], used for the 2011 IPF evidence-based guideline and again for the more recent 2015 IPF treatment update [10].

## Evidence-based Guideline Development

Some believe that the opinion of experts in the field is driven by their understanding of the clinical problem and their accurate interpretation of the underlying literature and therefore such recommendations may be considered, in effect, evidence-based. This approach has gone wrong on many occasions [11, 12]. Using a transparent and structured process in guideline development not only mandates a linkage of evidence to recommendation development but also ensures this linkage is explicit and systematically demonstrated [13–20]. In effect, this helps to limit the potential for bias. Guideline panel members may have strong opinions or academic bias surrounding a specific area in which they have clinical expertise or they may have other bias related to interactions with academic colleagues or industry partners [21, 22]. In an attempt to address this, GRADE requires systematic or pragmatic searches of the literature and the production of evidence summaries such as evidence profiles [23], ideally based on pooled treatment effects and produced by panel members without conflict of interest (COI) or independent methodologists. The latter helps to ensure a fair and reproducible assessment of the current literature addressing a specific clinical question [21].

Another significant benefit of evidence-based guidelines using GRADE methodology is the formal assessment of certainty in treatment effects (also known as quality of evidence or confidence in evidence). GRADE mandates a systematic and explicit assessment of specific methodological domains in order to evaluate how certain we are in the evidence for each outcome [24]. The certainty in treatment effect is then considered by the guideline panel and factored into the judgement regarding the strength and direction of recommendations. In this regard, several guidelines that followed unstructured approaches have unfortunately made inappropriately strong recommendations without considering the certainty in the evidence. As an example from the IPF literature, the 2000 consensus statement recommended treatment with azathioprine and corticosteroids for patients with IPF without explicit quality of evidence assessment [5]. Subsequent RCTs have since demonstrated the harm of this treatment intervention [25].

For the 2015 IPF treatment guideline, the McMaster University GRADE (MacGRADE; cebgrade.mcmaster.ca) team performed comprehensive systematic reviews for each of the 12 clinical questions. These were done in collaboration with clinical experts in the field to ensure proper question development and an experienced information scientist [26]. These systematic reviews provided the IPF guideline panel, including experienced IPF experts (who based on their involvement in IPF clinical trials and direct financial COI were considered conflicted panel members) and the non-conflicted members with the best available evidence summaries on which to base recommendations. The conflicted and non-conflicted members of the panel discussed all the evidence summaries in an open format, thereby enabling the non-conflicted panel members to deepen their understanding of the clinical relevance of the data and seek clarification as required. The evidence summaries were included as part of the guideline document to ensure the transparency of the entire process.

Certainties in evidence assessments were performed by the MacGRADE team and were then reviewed by the entire panel to ensure accuracy and agreement. In each case that the certainty in effect estimates was downgraded, explicit rationale was provided in the evidence profile and in the guideline manuscript. Including the certainty as part of the final recommendation, as we have done for the 2015 IPF evidence-based guideline, helps stakeholders in interpreting the recommendations made by the panel. Recommendations based on higher quality of evidence allow clinicians and patients to be more reassured that this intervention is beneficial. Recommendations based on lower quality of evidence provide caution to stakeholders and recognize the uncertainty that exists regarding the benefits of this intervention.

Although the estimate of treatment effect and certainty in evidence are important, guideline panelists should also consider other factors when deciding on the strength of recommendations. Elements such as the balance between the desirable and undesirable effects, the resources required, the impact on health equality, the acceptability and the feasibility of treatment must also be considered [20]. As opposed to consensus documents that use an *ad hoc* approach, GRADE mandates an explicit assessment of these criteria, using the Evidence-to-Decision framework (EtD), with documentation of panel judgements and rationale [19, 20, 27].

Input from clinical experts in the field is integral and guideline panelists must interpret the evidence summary, the certainty in estimate effects and consider the factors listed above in order to arrive at a direction and strength of recommendation using the EtD. Although some judgements will require an element of subjectivity, GRADE ensures explicit recording of rationale in order to improve transparency and reproducibility. Discussion of the evidence summaries and other EtD criteria for the 2015 IPF guidelines included all panel members, however, in order to help ensure impartiality, only non-conflicted members of the panel were permitted to formulate the recommendations themselves [22].

The final result, using the GRADE process, is a comprehensive, systematic, and explicit evidence-based guideline. Recommendations for the 2015 IPF update were formulated using the common terminology of “we suggest” for conditional (also known as weak) recommendations and “we recommend” for strong recommendations [19]. Strong recommendations are those that are applicable to the vast majority of patients, understanding a small minority will choose the opposite course of action. These recommendations are sometimes used to drive policy decisions. Conditional recommendations should apply to the majority of patients but there will be a large minority who will choose the opposite [13]. For conditional recommendations, especially those based on low or very low certainty of evidence, a model of shared decision-making between clinicians and patients is imperative considering all the factors above in addition to the individual patient’s values and preferences [28]. In essence, providing the recommendation in clear language along with a descriptive rationale empowers patients, clinicians, and stakeholders to better understand how the recommendations were formulated and to better apply them to their specific clinical practices and situations. This process using GRADE methodology is inherently different than that used by regulatory agencies when they consider market approval for pharmacological agents for the treatment of IPF.

## Conclusion

In summary, guidelines lacking the methodological components described above, especially those on topics with sufficient evidence, while conveying the (unstructured) opinions of clinical experts in the field, have a significant risk of providing biased recommendations that may be then used to guide patient care. With the increasing amount of evidence that has accumulated since the 2000 guidelines, the evolution towards evidence-based guidelines using the methodology described for a complex disease such as IPF is a clear benefit to all and represents a true advance in clinical science and patient-centered healthcare. At the end, what arguments can be made against transparency in guideline development?

## Abbreviations

ALAT: Latin American Thoracic Society
ATS: American Thoracic Society
COI: conflict of interest
ERS: European Respiratory Society
EtD: evidence to decision
GRADE: Grading of Recommendations Assessment, Development and Evaluation
IPF: Idiopathic pulmonary fibrosis
JRS: Japanese Respiratory Society
MacGRADE: McMaster University GRADE group
RCT: randomized controlled trials
